# Model to assess workload of village doctors in the National Essential Public Health Services Program in six provinces of China

**DOI:** 10.1186/s12913-020-05992-y

**Published:** 2020-12-09

**Authors:** Delu Yin, Tao Yin, Huiming Yang, Lihong Wang, Bowen Chen

**Affiliations:** 1 Capital Institute of Pediatrics, 2 YaBao Road, #330, ChaoYang District, Beijing, 100020 China; 2 Community Health Association of China, 2 YaBao Road, #330, ChaoYang District, Beijing, 100020 China

**Keywords:** Workload, Village doctors, National Essential Public Health Services program, Equivalent value

## Abstract

**Background:**

No studies, particularly quantitative analyses, have been conducted regarding the workload of village doctors in the National Essential Public Health Services (NEPHS) program and differences in service delivery by village doctors, according to region and services. In this study, we developed a quantitative analysis approach to measure the workload of NEPHS provided by village doctors in six provinces of China in 2016. We aimed to identify areas and services of the NEPHS needing improvement, so as to implement targeted measures to ensure adequate delivery of NEPHSs in rural remote underserved areas.

**Methods:**

Based on survey data from 300 town hospital centers (THCs) located in 60 counties in the six selected provinces, we calculated village doctors’ share of workload under the NEPHS using the equivalent value (EV) model. To define the workload and corresponding EV of each NEPHS, a series of five meetings was held with THC managers, public health workers, family physicians, nurses and village doctors. Field observations were conducted to verify the workload and EV of each service.

**Results:**

Village doctors’ share of the workload under the NEPHS program was 43.71% across the 300 sampled THCs in six provinces. The village doctors’ workload shares for different NEPHS ranged from 17.14 to 57.00%. The percentage workload undertaken by village doctors under the NEPHS program varied across different provinces, with the highest proportion 63.4% and the lowest 28.5%.

**Conclusions:**

The total NEPHS workload assigned to village doctors by THCs in the six sampled provinces exceeded the Chinese government’s requirement of 40%, but the workload proportion in some provinces was less than 40%. In addition, the percentage workload for some NEPHS undertaken by village doctors was lower than others. We suggest conducting district-level analysis of the workload among village doctors under the NEPHS program using the EV method, to identify areas and services needing improvement, to implement targeted measures to expand and promote health service provision in China’s rural underserved areas.

**Supplementary Information:**

The online version contains supplementary material available at 10.1186/s12913-020-05992-y.

## Background

Recent decades have witnessed rapid improvement in China’s public health system, with average life expectancy up from 71.4 to 74.8 years between 2000 and 2010 [1, 2], the maternal mortality rate down from 53.0 to 30.0 per 100,000 population and the infant mortality rate dropping from32.2 to 13.1 per 1000 live births between 2000 and 2010 [3–5]. Despite this progress, diseases including hypertension, obesity, diabetes and other non-communicable diseases still pose challenges to China’s public health [6, 7]. To tackle these issues and rid patients of unnecessary and costly secondary and tertiary care, early prevention and intervention programs have been in place since 2009. One of these, called the National Essential Public Health Services (NEPHS) program, is intended to provide equal access to public health services regardless of geographic location and to expand the coverage of essential public health services to all Chinese residents [8]. The NEPHSs are the most essential public health services provided free of charge to all residents, focusing on children, pregnant women, the elderly and patients with chronic diseases, aiming at the main health problems of urban and rural residents. The funds needed to provide these services are mainly borne by the governments. With the economic and social development, changes of public health service needs and financial affordability, the NEPHS items will be adjusted timely [9]. The NEPHS program aims to narrow the gap between rural and urban residents with respect to essential public health services and to ensure equity of access to these services [9]. Since the NEPHS program was launched 10 years ago, the types of services included in this program have progressively increased, from nine in 2009 to 12 in 2016. The allowance standard per capita for the program has increased from 2.38 USD in 2009 to 7.94 USD in 2016. The 12 types of public health services mainly target children aged 0–6 years, pregnant women, those aged over 65 and patients with mental health issues and chronic conditions [10, 11]. Services are offered through community-based primary health care facilities [9]. These are universally accessible and cover town hospital centers (THCs) and village clinics serving 589.7 million residents in rural areas of China as well as community health service facilities that served 793.0 million residents in urban areas in 2016 [12]. In particular, the NEPHS program in China differs from those outlined by the World Health Organization [13] and by the US Centers for Disease Control and Prevention [14]. China’s NEPHS are only provided by primary health care facilities, not the whole public health system. Secondly, the program focuses on essential primary care (maternal, infant and older adult health) accessible for all people, rather than regulatory and monitoring tasks.

Chinese healthcare systems covering both urban and rural residents have been put in place. In the rural areas, it refers to a three-level medical service network that comprises the county hospital, the THCs and village clinics, with the county hospital performing the leading role, and THCs and village clinics service at the base. And in the cities and towns, it refers to a new type of urban medical health service system that features division of responsibilities as well as cooperation among various types of hospitals at all levels and community healthcare centers [15, 16]. This rural three-tiered system was designed to promote the efficient allocation of health care resources between primary and tertiary care facilities. In the system, county hospitals, THCs, and village clinics differ in scale and functions. Ideally, patients with minor ailments are directed to village doctors in village clinics (primary facilities). Patients with more serious illnesses are transferred to THCs (second-tier or secondary facilities), and only the most seriously ill are sent to county hospitals (third-tier or tertiary facilities) [15]. THCs generally have different numbers of inpatient beds (35.35 on average in 2016 [17]) whereas village clinics have no beds. Chinese rural doctors were originally called “barefoot doctors”, born in the 1950s. They referred to the rural medical personnel who were not formally trained in medical treatment, still hold agricultural household registration, and in some cases were “semi agricultural and semi medical” [18]. China implemented the “Regulations on the Administration of Village Doctors” to improve the professional quality of rural doctors on 1 January 2004. Village doctors are now required to finish 2 to 3 years of professional training. Only village doctors who pass the professional training and corresponding examination can obtain the practice certificate [18]. As the basis of health service networks in rural areas, village doctors currently serve nearly half the population of China (42.6%). Before the start of the NEPHS program, there were 864,000 village doctors working in 583,000 village clinics in 2005. By the end of 2016, 639,000 village clinics had been set up in 560,000 administrative villages across China. In total, there are 1,436,000 staff members in these village clinics, including 320,000 practicing (assistant) physicians and 933,000 village doctors. The number of consultations provided by village doctors and practicing (assistant) physicians in 2016 reached 1.85 billion, 2.57 times that of consultations provided to urban residents by practitioners working in community health service facilities (0.72 billion) [19]. The number of residents served by one village doctor varies with the number of doctors in the village. On average, one village doctor might provide primary health care for 1000–2000 rural residents [19].

It is mandatory for township hospital centers (THCs) and village clinics to provide NEPHS to rural residents [20]. Central and local governments are responsible for subsidizing village doctors for providing these services [21]. The willingness of village doctors and THC managers to provide NEPHS is relatively high because of the introduction of a minimum subsidy [12]. The NEPHS program allowance makes up the largest proportion of village doctors’ income throughout most of rural China [12, 22]. In an effort to reasonably distribute the total workload among THCs and village clinics, the government also transfers, in principle, no less than 40% of the NEPHS workload to village doctors via the program allowance in the form of government service purchases based on performance assessment since 2016 [23]. The central government encourages village doctors to undertake a greater workload in the NEPHS program, to ensure income for village doctors from NEPHS. However, no ideal national workload standard for village doctors in the NEPHS has been developed owing to great differences among rural regions of China [23]. Funds are paid to the THCs and allocated by the THCs to village doctors and THCs. The distribution is based on the estimate of village doctors’ performance generally including work volume, quality and residents’ satisfaction with NEPHS [12]. THCs might impose an economic penalty on village doctors for work that is poor or unfinished [20, 24].

It is widely accepted that medical and health services in rural China are insufficient. Village doctors have an increasingly important role in the provision of NEPHS in rural China [24, 25]. The government has attached great importance to facilitating the provision of a greater quantity and higher quality of NEPHS by village doctors, which not only helps to improve the accessibility to public health services in rural areas but also stabilizes teams of village doctors [25, 26]. In recent years, several qualitative studies have been carried out on the provision of NEPHS by village doctors [12, 18, 24–26]. The study findings show that these doctors face many challenges in providing NEPHS and many factors affect the provision of health services. Some factors like village doctors’ age, sex (in most districts of China, male village doctors do not provide maternal health services to women), or poor level of education are difficult to change whereas others can be improved, such as lack of effective incentives, poor computer skills, inadequate professional NEPHS training, insufficient equipment allocation and lack of cooperation from rural residents [12, 18]. Identifying areas and services of NEPHS that need improvement, so as to adopt targeted measures to expand these, is conducive to ensuring the adequate delivery of NEPHS in rural remote areas. It is a challenge for primary health managers, some of whom are not medical professionals, to identify weak areas and services within the NEPH program unless simple quantitative methods and tools are used. However, there are no studies, particularly quantitative analyses, on the public health service workload of village doctors and differences in service delivery by village doctors according to region and services. The present analysis can provide a comprehensive perspective on the provision of public health services in rural areas by village doctors. We used a model-based method to calculate the NEPHS workload of village doctors in 300 THCs in 60 counties of six provinces in China. Our findings can provide a reference point and basis for governments at all levels, especially grass-roots health management departments, to identify areas and services of the NEPHS program needing improvement, to implement measures to expand services in underserved areas.

## Methods

### Sampling

In this study, we adopted a random cluster sampling method. Among six sample provinces, two provinces were selected for this survey in the eastern, central and western regions (Eastern: Zhejiang and Fujian; central: Anhui and Henan; western: Yunnan and Shaanxi). In each province, we chose five prefecture-level cities at random and in each prefecture-level city, we randomly selected two counties. Five THCs were randomly chosen from each county. In total, 300 THCs from six provinces were surveyed. On average, each THC has 40 on-duty staff, 34 health care specialists, 16 doctors and 5 certified (assistant) doctors, with an average of 37,270 outpatient and emergency visits plus 1344 inpatients per year. An average of 20 administrative villages and 16 village clinics are under the governance of each THC. Each village clinic is staffed by 1.3 health care professionals.

### Data collection

Using the NEPHS guidelines for 2011, the research team designed questionnaires and survey instructions (see the questionnaire on job responsibilities of village doctors in the supplementary material). Survey questions covered basic information of the sampled THCs and statistics of the total workload assigned according to the 12 types of services and the share of workload undertaken by village doctors in village clinics. The share of workload undertaken by village doctors in village clinics was estimated and reported separately by each service-related department in THCs according to the actual workload carried out by village doctors. The survey tool was pre-tested in two THCs of Beijing and further revised. Before conducting the survey, the research team confirmed the list of sample provinces, cities and counties. To guarantee a high response rate, the Primary Health Department of the National Health and Family Planning Commission issued a notice regarding the survey. Provincial health and family planning administrative departments in the sample provinces organized sample counties and selected sample THCs, as required, checking whether all answers on the questionnaires had been completed and reviewing the data for accuracy. After receiving the questionnaires, the research team rechecked everything and carried out logic checks. During the survey, the research team provided advice by telephone and kept records.

### Model

The equivalent value (EV) method has been used to estimate the cost of the NEPHS program and to calculate community health-staffing requirements [27–31]. In the present study, we used the EV method to build a model for measuring the workload of NEPHS provided by village doctors, according to the following steps: 1) determine the standard service protocol of all types of NEPHS; 2) determine the workload and EV of each NEPHS compared with a standard clinic visit; 3) calculate the village doctors’ workload in the NEPHS program.

#### Step 1: determining the standard service protocol

In 2016, the NEPHS program had 12 types of public health services. These included establishing health records for residents; management of patients with chronic non-communicable diseases; physical examination for major diseases in children, women and older people; health education; vaccination services for vaccine-preventable diseases and prevention and control of major infectious diseases. All 12 types of NEPHS are included in the 2011 NEPHS guidelines [11].

#### Step 2: determining the workload and EV of each NEPHS

To determine workload (person-time), we used a multistage iterative feedback and revision process [30–32]. Participants (*n* = 60) from the six sampled provinces were invited to attend a series of five meetings according to their expertise with NEPHS. Participants included THC managers (*n* = 12), public health workers (*n* = 12), family physicians (*n* = 12), nurses (*n* = 12) and village doctors (*n* = 12). During the meetings, participants discussed the amount of person-time required for each NEPHS, according to the 2011 NEPHS guidelines. Participants also suggested modifications to the workload indicators. Socioeconomic education levels among villagers as well as population density in western provinces are lower than those in the central and eastern provinces. Participants generally believed that these differences affect the delivery of NEPHS, thus affecting their workload [30–32]. For example, for rural residents in western China, a clinic visit or home visit may require more time from village doctors. Additionally, NEPHS may be provided using mobile medical facilities to assure service accessibility, which will also have an impact on the NEPHS workload. The gap in the workload of NEPHS between eastern and central China was relatively small, so these were combined into one region, and two sets of specific workload were created for each NEPHS.

To test the workload of each NEPHS, four research assistants were trained to observe and record the person-time for each type of service in 12 THCs randomly chosen from the six sampled provinces. In terms of services that could not be recorded during direct observation, face-to face interviews with public health workers were conducted to determine their workload. The person-time for each NEPHS was rechecked and modified on the basis of direct observation and interviews.

To ensure that different public health services could be compared directly, a “standard clinic visit” was introduced as a benchmark to gauge the EV for NEPHS [27–32]. A standard clinic visit referred to a family physician consulting with one patient for 15 min [33] and the EV of a standard clinic visit was defined as 1. The EV of each NEPHS was then determined based on the person-time compared with a standard clinic visit. The workload and EV of each NEPHS in different areas was defined separately and is shown in Table 1.

**Table 1:** Workload and EV of each NEPHS in different areas (compared with a standard clinic visit)

Types	Service Contents	Unit	Mid-east China^a^		Western China	
			Workload (minutes)	Mean EV	Workload (minutes)	Mean EV
Standard clinic visit	Family physician consulting with patient	per person	15.0	1.0	15.0	1.0
Health records management service	Establish residents’ health records	per person	30.0	2.0	37.5	2.5
	Update of residents’ health records	per person	7.5	0.5	12.0	0.8
Health education	Make annual implementation plan of health education	per time	720.0	48.0	1170.0	78.0
	Set up health education bulletin board	per time	373.5	24.9	720.0	48.0
	Public health consultation activities	per time	669.0	44.6	135.0	9.0
	Public health knowledge lecture	per time	463.5	30.9	480.0	32.0
Immunizations	Establish vaccination file	per person	9.0	0.6	10.5	0.7
	Vaccination service	per visit	16.5	1.1	16.5	1.1
	Handling of suspected abnormal vaccination reaction	per visit	18.0	1.2	6.0	0.4
Health services for children aged 0 to 6 years	Family visit of newborn	per visit	118.5	7.9	52.5	3.5
	42 days follow-up	per visit	72.0	4.8	51.0	3.4
	Infant physical examination	per visit	30.0	2.0	16.5	1.1
	Physical examination of preschool children	per visit	30.0	2.0	69.0	4.6
Maternal health services	Early pregnancy health management	per visit	54.0	3.6	37.5	2.5
	Health management in the second trimester	per visit	40.5	2.7	27.0	1.8
	Health management in late pregnancy	per visit	42.0	2.8	27.0	1.8
	Postpartum visit	per visit	81.0	5.4	30.0	2.0
	42 days postpartum health examination	per visit	42.0	2.8	28.5	1.9
Elderly people’s health services	Physical examination for the elderly	per person	39.0	2.6	34.5	2.3
	Health guidance for the elderly	per person	12.0	0.8	6.0	0.4
Health services for patients with hypertension	Screening of patients with hypertension	per person	39.0	2.6	24.0	1.6
	Follow up evaluation and classified intervention of hypertension patients	per visit	36.0	2.4	36.0	2.4
	Health examination for patients with hypertension	per person	42.0	2.8	34.5	2.3
Health services for patients with type II diabetes	Diabetes screening	per person	42.0	2.8	31.5	2.1
	Follow up evaluation and classified intervention of diabetic patients	per visit	36.0	2.4	37.5	2.5
	Physical examination of diabetic patients	per person	42.0	2.8	37.5	2.5
Health services for patients with severe mental illness	Information management of patients with severe mental illness	per person	108.0	7.2	79.5	5.3
	Follow up evaluation and classified intervention of severe mental illness	per visit	72.0	4.8	42.0	2.8
	Physical examination for severe mental illness	per person	66.0	4.4	43.5	2.9
Reporting and management of infectious diseases and public health emergencies	Discovery and registration of infectious diseases and public health emergencies	per time	114	7.6	21.0	1.4
	Report and handling report of infectious diseases and public health emergencies	per time	121.5	8.1	7.5	0.5
Health management with Chinese medicine	Recognition of TCM constitution (aged over 65)	per visit	30.0	2.0	27.0	1.8
	Health care guidance of traditional Chinese medicine (over 65 years old)	per visit	15.0	1.0	13.5	0.9
	Chinese medicine health guidance for children (6, 12, 18, 24, 30, 36 months old)	per visit	24.0	1.6	21.0	1.4
Health supervision assistance services	Food safety information report of health supervision	per time	565.5	37.7	495.0	33.0
	Occupational health consultation guidance	per time	205.5	13.7	180.0	12.0
	Health supervision assists in the inspection of drinking water health and safety	per time	514.5	34.3	517.5	34.5
	Health supervision and coordination of school health services	per time	171.0	11.4	90.0	6.0

Abbreviations: *NEPHSP* National Essential Public Health Services program, *EV* equivalent value, *TCM* traditional Chinese medicine

^a^ The gap in the NEPHS EV between eastern and central China was relatively small, so these are combined into one region in the table

#### Step 3: calculate village doctors’ workload in the NEPHS program

Based on the EV of each NEPHS, the workload undertaken by village doctors under the NEPHS program was calculated using the following process:

$$ \mathbf{Share}\ \mathbf{of}\ \mathbf{the}\ \mathbf{workload}\ \mathbf{under}\mathbf{taken}\ \mathbf{by}\ \mathbf{village}\ \mathbf{doctors}\ \mathbf{under}\ \mathbf{the}\ \mathbf{NEPHS}\ \mathbf{program}=\mathrm{EVs}\ \mathrm{of}\ \mathrm{services}\ \mathrm{performed}\ \mathrm{by}\ \mathrm{village}\ \mathrm{doctors}\ \mathrm{under}\ \mathrm{the}\ \mathrm{NEPHS}\ \mathrm{program}\ \left(\mathrm{X}\right)/\mathrm{total}\ \mathrm{EVs}\ \mathrm{of}\ \mathrm{services}\ \mathrm{in}\mathrm{cluded}\ \mathrm{in}\ \mathrm{the}\ \mathrm{NEPHS}\ \mathrm{program}\ \mathrm{provided}\ \mathrm{by}\ \mathrm{sampled}\ \mathrm{THCs}\ \left(\mathrm{Y}\right)\times 100\% $$

$$ \mathbf{Y}=\sum \mathrm{EV}\ \mathrm{of}\ \mathrm{each}\ \mathrm{public}\ \mathrm{health}\ \mathrm{service}\ \mathrm{item}\ \left(\mathrm{A}\right)\times \mathrm{volume}\ \mathrm{of}\ \mathrm{each}\ \mathrm{public}\ \mathrm{health}\ \mathrm{service}\ \mathrm{item}\ \left(\mathrm{B}\right) $$

$$ \mathbf{X}=\sum \mathrm{EV}\ \mathrm{of}\ \mathrm{each}\ \mathrm{public}\ \mathrm{health}\ \mathrm{service}\ \mathrm{item}\ \left(\mathrm{A}\right)\times \mathrm{volume}\ \mathrm{of}\ \mathrm{each}\ \mathrm{public}\ \mathrm{health}\ \mathrm{service}\ \mathrm{item}\ \left(\mathrm{B}\right)\times \mathrm{village}\ \mathrm{doctors}'\mathrm{share}\ \mathrm{of}\ \mathrm{workload}\ \mathrm{for}\ \mathrm{each}\ \mathrm{public}\ \mathrm{health}\ \mathrm{service}\ \mathrm{item}\ \left(\mathrm{C}\right) $$

**A:** sourced from the EV of each NEPHS in the Table 1;

**B:** sourced from surveys on the total workload assigned according to the 12 types of service (see section 2.2, Data Collection);

**C:** sourced from surveys regarding the share of workload undertaken by village doctors in village clinics. This was estimated and reported separately by each service-related department of THCs according to the actual workload conducted by village doctors (see the Data Collection section).

## Results

### Workload for different NEPHS provided by village doctors

The total workload of NEPHS was 42.32 million EVs across 300 sample THCs from six provinces; village doctors carried out 43.71% of the total workload under the NEPHS program in 2016. The workload share of different NEPHS undertaken by village doctors ranged from 17.10 to 57.00%; the workload share was more than 50.00% of health services for patients with hypertension, type II diabetes and severe mental illness whereas it was relatively lower for other services including reporting and management of infectious diseases and public health emergencies, health supervision assistance services and immunizations (Table 2).

**Table 2:** Workload of different NEPHS provided by village doctors (2016)

Types of essential public health services	Total EVs undertaken by sampled THCs (One Millions)	EVs Undertaken by village doctors (One Millions)	Share of workload undertaken by village doctors (%)
Health records management service	6.59	3.27	49.54
Health education	7.13	3.37	47.24
Immunizations	3.86	1.10	28.42
Health services for children aged 0 to 6 years	6.03	1.86	30.91
Maternal health services	1.40	0.45	31.83
Elderly people’s health services	4.27	1.78	41.80
Health services for patients with hypertension	7.35	4.19	57.00
Health services for patients with type II diabetes	1.98	1.10	55.42
Health services for patients with severe mental illness	0.68	0.35	51.76
Reporting and management of infectious diseases and public health emergencies	0.12	0.02	17.14
Health management with Chinese medicine	2.08	0.80	38.43
Health supervision assistance services	0.84	0.22	26.20
**Total**	42.32	18.50	43.71

Abbreviations: *NEPHS* National Essential Public Health Services, *EV* equivalent value, *THC* town hospital center

### Percentage workload of different NEPHS provided by village doctors in different provinces

Our comparative analysis showed that the percentage workload undertaken by village doctors under the NEPHS program varied among different provinces, with the highest proportion 63.4% and the lowest 28.5%. The workload share for different types of public health services provided by village doctors also varied in different provinces (Table 3). To make some proposals regarding the reasons underlying this variation, the number of village clinics, health workers, and village doctors in the six provinces were compared [17]. Preliminary comparative analysis showed that provinces with a higher average number of health workers per village clinic (provinces 4 and 6) had a higher proportion of the NEPHS workload carried by village doctors.

**Table 3:** Percentage workload undertaken by village doctors in different provinces (2016), NEPHSP

Types of essential public health services	Province1	Province2	Province3	Province4	Province5	Province6
Health records management service	26.6	48.1	34.9	78.0	52.6	49.4
Health education	35.3	45.6	28.0	59.6	47.2	64.2
Immunizations	34.4	23.1	21.6	26.6	27.8	33.5
Health services for children aged 0 to 6 years	27.6	28.9	37.5	30.2	30.9	30.0
Maternal health services	29.7	34.2	37.9	27.6	24.1	36.1
Elderly people’s health services	44.5	42.8	32.1	48.0	46.3	39.9
Health services for patients with hypertension	53.4	64.2	60.5	59.0	55.3	49.8
Health services for patients with type II diabetes	48.1	55.0	57.2	53.1	55.6	62.0
Health services for patients with severe mental illness	55.8	46.5	51.3	48.1	53.8	54.6
Reporting and management of infectious diseases and public health emergencies	17.9	18.8	14.3	18.6	22.6	13.2
Health management with Chinese medicine	40.0	34.0	39.3	36.7	41.5	38.6
Health supervision assistance services	19.1	41.6	21.3	17.8	18.6	41.0
**Total**	**35.5**	**45.5**	**28.5**	**59.2**	**47.1**	**63.4**

Abbreviations: *NEPHSP* National Essential Public Health Services program

## Discussion

In this study, we carried out quantitative analysis of the workload of village doctors under the NEPHS program and calculated the share of the total workload among these doctors. Our results showed that 43.71% of the total NEPHS workload was assigned to village doctors by THCs in the six sampled provinces, exceeding the Chinese government’s requirement of 40%. There were differences in the percentage NEPHS workload undertaken by village doctors among different provinces and according to different types of service. The reasons for these differences are unclear and require further research. However, previous studies have shown that shortages and aging of village doctors are two of the main barriers to providing public health services among village doctors. Insufficient training and shortages of necessary medical equipment also restrict village doctors’ ability to provide additional services [18, 24]. Moreover, establishment of unreasonable performance indicators by THCs and unbalanced allowance quotas has largely affected the enthusiasm of village doctors to provide these services [12, 18]. Consequently, differences among the six sampled provinces may be attributed to the number and capabilities of village doctors, performance indicators, allowance quotas for village doctors and other unknown factors [12, 24, 25]. The workload undertaken by village doctors in the NEPHS program varies according to type of service, which may be related to village doctors’ age, sex, computer skills and the number of nurses and administrative staff available to support village doctors [18].In this study, preliminary comparative analysis showed that the percentage workload undertaken by village doctors may be affected not only by the number of village doctors but also the number of other health workers in clinics, such as nurses, because these health workers can undertake some NEPHS tasks.

The present results generate some possible directions for policy adjustment. It is vital to further strengthen the important role of village doctors in providing NEPHS, to guarantee the availability of these services in rural areas. This study showed that differences exist in the percentage of the NEPHS workload taken on by village doctors according to different provinces and different services, with the percentage workload in some provinces lower than 40%. In terms of the types of services and provinces with a lower share of the workload, governments should focus on those factors can be changed and take appropriate steps, including improving professional skills among village doctors by intensifying professional training, equipping village doctors with the necessary medical facilities and equipment and encouraging them to undertake more work under the NEPHS program by improving performance evaluation and granting a higher program allowance [12, 18]. The EV method is simple and easy, such that primary health management departments can identify weak areas and services by collecting general data to input in the model. This provides a reference point and basis for identifying areas and services of the NEPHS program needing improvement, so that measures to expand services in underserved areas can be implemented. Therefore, to tackle the question of whether the current workforce can adequately deliver NEPHS in rural areas, it is suggested that government at all levels, especially grass-roots health management departments, adopt the EV method to identify priority areas and services in rural regions, by estimating the proportion administered by village doctors. In addition, the EV method can be used to measure the role of village doctors in providing all primary health services as a whole, so as to target measures for expanding services and improve the role of village doctors as health gatekeepers in rural remote areas.

This study is the first in China to calculate the workload proportion of village doctors in the NEPHS program. In recent years, relevant documents issued by the government continue to emphasize the basic requirement of 40% [23]. The proportion exceeding the service capacity of village doctors is unfavorable to the stability of these doctors as well as to service provision. We should note that China’s policy of public health service equalization means that the workload among village doctors is increasing [24]. We must ensure that the workload assigned to these doctors is manageable, based on their individual capabilities [34]. Workloads should not be blindly increased, to avoid undermining rural residents’ access to quality public health services [20, 35, 36]. In our study, the percentage workload undertaken by village doctors in two provinces was more than 60%. We therefore suggest that local governments monitor village doctors’ workload in the NEPHS program using the model developed in this study.

Previous studies have shown that THCs and village doctors have different opinions regarding the workload share and job responsibilities allocated to doctors [37, 38]. The measurement in this study may underestimate the workload of village doctors in the NEPHS program. Therefore, a limitation of this research is that we did not investigate workloads from the perspective of doctors in the village clinics themselves. In future, calculations and comparative analyses should be conducted from the perspective of village doctors working in the NEPHS program.

## Conclusions

It is vital for primary health managers to identify areas and services of the NEPHS program that require improvement and to adopt targeted measures to expand these, so as to ensure the adequate delivery of NEPHS in rural remote areas. However, no studies or quantitative analyses to date have investigated the workload of village doctors in the NEPHS program and differences in service delivery by village doctors according to region and services. In this study, we used a quantitative analysis approach and found that 43.71% of the total NEPHS workload was assigned to village doctors by THCs in six sampled provinces, exceeding the Chinese government’s requirement of 40%. Differences exist in the percentage of the NEPHS workload undertaken by village doctors among different provinces and services, with the workload proportion in some provinces less than 40%.We suggest conducting district-level analysis of the workload among village doctors under the NEPHS program using the EV method in China’s rural underserved areas, to identify areas and services of the NEPHS program that require improvement and implement targeted measures to promote health service provision. The present model based on EV of the NEPHS could be used to monitor village doctors’ workload under the NEPHS program as well to ensure that the workload assigned to village doctors is manageable.

## Supplementary Information

**Additional file 1.** Questionnaire on workload of village doctors in the National Essential Public Health Services Program (NEPHSP).

## Data Availability

Questionnaire on the job responsibilities of village doctors is provided as Supplementary file 1.

## Abbreviations

NEPHSP: National Essential Public Health Services Program
THC: Township Hospital Centers
EV: Equivalent value
