# Supplementary material for: Model to assess workload of village doctors in the National Essential Public Health Services Program in six provinces of China
Source: BMC Health Serv Res. 2020 Dec 9;20:1134. doi: 10.1186/s12913-020-05992-y (PMC7727222; doi:10.1186/s12913-020-05992-y)
Supplement: Supplementary file 1 — Additional file 1. Questionnaire on workload of village doctors in the National Essential Public Health Services Program (NEPHSP). [file 12913_2020_5992_MOESM1_ESM.docx]

**Questionnaire on the job responsibilities undertaken by village doctors under the National Essential Public Health Services Package (NEPHSP)**

| Name of township health center: ______________________ | | | | | | |
| --- | --- | --- | --- | --- | --- | --- |
| Address: ______________________ | | | | | | |
| Items | | |  | | | |
| Population of the township health centers | | |  | | | |
| Volume of Outpatient and Emergency visits | | |  | | | |
| Volume of Inpatients | | |  | | | |
| Number of the on-duty staff | | |  | | | |
| Number of the healthcare specialists | | |  | | | |
| Number of the affiliated administrative villages | | |  | | | |
| Number of the affiliated administrative village clinics | | |  | | | |
| Number of the healthcare specialists in village clinics | | |  | | | |
| Allowance received by the township health center | | |  | | | |
| Allowance received by village doctors | | |  | | | |
| Job responsibilities undertaken by village doctors under the National Essential Public Health Services Package (NEPHSP) | | | Total Volume | | Workload Estimate undertaken by village doctors（%） | |
| 1.Resident health records management | | 1.Establishing health records* |  | |  | |
|  |  | 2.Maintaining and managing health records（using）* |  | |  | |
| 2.Health education | | 1.Providing health education materials* |  | |  | |
|  |  | 2.Designing health education bulletin boards* |  | |  | |
|  |  | 3.Offering consultation services on public health* |  | |  | |
|  |  | 4.Holding lectures on health knowledge* |  | |  | |
|  |  | 5.Carrying out individualized health education* |  | |  | |
| 3.Immunizations | | 1.Staffing* |  | |  | |
|  |  | 2.Immunization management |  | |  | |
|  |  | 3.Immunizations |  | |  | |
|  |  | 4.Handling suspected adverse events following immunizations |  | |  | |
| 4.Health services for children aged 0 to 6 years | | 1.Staffing and information management* |  | |  | |
|  |  | 2.Home visits to newborn infants* |  | |  | |
|  |  | 3.Health services for newborn infants who are just over one month old* |  | |  | |
|  |  | 4.Health services for infants |  | |  | |
|  |  | 5. Health services for preschool-age children* |  | |  | |
| 5.Maternal health services | | 1.Staffing and information management |  | |  | |
|  |  | 2.Maternity care during early pregnancy |  | |  | |
|  |  | 3.Maternity care during mid-pregnancy* |  | |  | |
|  |  | 4.Maternity care during late-pregnancy |  | |  | |
|  |  | 5.After-delivery visits* |  | |  | |
|  |  | 6.Post-natal physical examination 42 days after delivery* |  | |  | |
| 6.Elderly people’s health services | | 1.Staffing and information management* |  | |  | |
|  |  | 2.Lifestyle and health conditions evaluation* |  | |  | |
|  |  | 3.Physical examinations |  | |  | |
|  |  | 4.Auxiliary examinations |  | |  | |
|  |  | 5.Health guidance* |  | |  | |
| 7.Health services for patients with hypertension | | 1.Screening* |  | |  | |
|  |  | 2.Following-up evaluation and classified interventions* |  | |  | |
|  |  | 3. Physical examinations* |  | |  | |
| 8.Health services for patients with type II diabetes | | 1.Screening* |  | |  | |
|  |  | 2. Following-up evaluation and classified interventions* |  | |  | |
|  |  | 3. Physical examinations |  | |  | |
| 9.Services for patients with severe mental illness | | 1.Managing health records for patients with severe mental illness |  | |  | |
|  |  | 2. Following-up evaluation and classified interventions |  | |  | |
|  |  | 3. Physical examinations |  | |  | |
| 10.Reporting and management of infectious diseases and public health emergencies | | 1.Risk management of infectious diseases and public health emergencies |  | |  | |
|  |  | 2.Discovering and registering infectious diseases and public health emergencies* |  | |  | |
|  |  | 3.Reporting information on infectious diseases and public health emergencies |  | |  | |
|  |  | 4.Responding to infectious diseases and public health emergencies |  | |  | |
| 11.Health management with Chinese medicine | | 1.Identifying constitutions for elderly people based on Chinese medicine* |  | |  | |
|  |  | 2.Paediatric aftercare with Chinese medicine* |  | |  | |
| 12.Health supervision assistance services | | 1.Report on food safety information* |  | |  | |
|  |  | 2.Consultation and guidance on occupational health |  | |  | |
|  |  | 3.Drinking water safety inspections |  | |  | |
|  |  | 4.School hygiene services* |  | |  | |
|  |  | 5.Report on illegal medical practices as well as illegal blood collection and supply* |  | |  | |
